# Can mathematical modelling work as a creativity-demanding activity? An empirical study in China

**DOI:** 10.1007/s11858-021-01316-4

**Published:** 2021-10-18

**Authors:** Xiaoli Lu, Gabriele Kaiser

**Affiliations:** 1 School of Mathematical Sciences and Shanghai Key Laboratory of PMMP, East China Normal University, Shanghai, China; 2 University of Hamburg, Hamburg, Germany; 3 Australian Cathollic University, Brisbane, Australia

## Abstract

Creativity has been identified as a key characteristic that allows students to adapt smoothly to rapid societal and economic changes in the real world. However, Chinese students appear to perform less well in mathematical problem-solving and problem-posing abilities, which are strongly connected to mathematical creativity. Mathematical modelling has recently been introduced as one of the six core competencies in the Chinese mathematical curriculum and is built on students’ ability to solve real-world problems using mathematical means. As mathematical modelling is characterised by openness regarding the understanding of complex real-world problems and the complex relationship between the real world and mathematics, for the strengthening of creativity, mathematical modelling activities seem to be adequate to accomplish this purpose. In this paper, we describe a study with 71 upper secondary school students, 50 pre-service mathematics teachers, and 66 in-service mathematics teachers, based on an extended didactical framework regarding mathematical modelling as a creativity-demanding activity. The results of the study indicate a significant correlation between modelling competencies and creativity aspects. Especially significant correlations between the adequacy of the modelling approaches and the two creativity aspects of usefulness and fluency could be identified, as well as a significant negative correlation between usefulness and originality. The results of the correlational analysis of relationships among the four criteria were not always consistent in the three participant groups. Overall, the results have implications for the promotion of creativity for various expertise groups and demonstrate the dependency of the modelling activities on the mathematical knowledge of the participants and the mathematical topic with which they are dealing.

## Introduction

Amid recent dramatic changes in work and life, organisations worldwide have independently developed the framework of twenty-first-century skills to promote education for responsible citizenship, and twenty-first-century learning has been advocated for in many national curricula (Binkley et al., 2012). Creativity has been considered a key twenty first-century skill (Wegerif & Dawes, 2004), including among others critical thinking, problem-solving, decision-making, communicating, collaborating, and information literacy (Maaß et al., 2019). However, these notions of twenty-first-century skills—particularly creativity—are usually included in the overarching statements of educational ideas or goals, and detailed descriptions or clearly elaborated frameworks to implement them are lacking (Binkley et al., 2012). A similar situation can be found in China’s newly released national curricular standards of mathematics, which—in line with the educational system’s overall aims—call for the core competencies to be developed by the students (Wang & Lu, 2018). Mathematics education has become part of citizenship education, owing to its strong relationship to twenty-first-century skills; specifically, a mathematical modelling perspective offers opportunities to promote these skills due to its potential contributions to addressing socio-economic issues and inspiring student-centred learning (Maaß et al., 2019).

Teachers play a central role in promoting student-centred learning (Leikin & Elgrably, 2020); moreover, effective teacher education programmes and supports for early-career teachers are necessary (Lu et al., 2021). Specifically, to develop students’ creativity, teachers must create adequate opportunities (Zazkis & Holton, 2009), and prospective teachers must develop their own creativity with the support of teacher educators (Zazkis, 2017). Lee (2017) highlighted the importance of promoting teachers’ profound mathematical knowledge, their competence in designing or modifying tasks to develop creativity, awareness, and positive dispositions toward creativity education, and the potential to combine teaching creativity and creative skills. China’s mathematical curriculum has a long history of emphasising teachers’ profound mathematics knowledge (Leung, 2001). Nowadays, the Ministry of Education of China emphasises the necessity of establishing a high-quality, professional, and creative teacher team (MOE, 2020), which requires that teacher education should go beyond establishing knowledge bases, in order to focus on creativity, such as by creative uses of new educational theoretical frameworks (Leikin & Elgrably, 2020).

Departing from the important role of mathematical modelling, which is emphasised in the recent national curricular standards for upper secondary school mathematics in China (MOE, 2018), in this study we aim to investigate the possibility of combining creativity and mathematical modelling to promote the development of twenty-first-century skills in the teaching and learning of mathematics within the context of Chinese education and beyond.

## Literature survey, theoretical framework, research context and research questions

### Creativity and its promotion

Research interest in creativity has increased in the last three decades, with its focus shifting from genius to wider perspectives of inquiry, such as creative behaviours in daily life (Hersh & Jone-Steiner, 2017). Creativity, critical thinking, problem-solving, decision-making, communication, collaborating, and information literacy are considered to be key twenty-first-century skills for promoting active citizenship (Maaß et al., 2019); in particular, creativity is one of the central cognitive skills that teachers and students should develop (Pellegrino & Hilton, 2012).

Mathematical creativity has been investigated specifically in relation to mathematical learning (Pitta-Pantazi et al., 2018). On the one hand, it is strongly related to general creativity—for example, general creativity is a prerequisite for mathematical creativity (Hong & Milgram, 2010)—and both share similar frameworks or evaluation methods (e.g., Pitta-Pantazi et al., 2018; Silver, 1997). On the other hand, mathematical creativity cannot be isolated as one aspect of general creativity owing to its domain-specificity (Kattou et al., 2015). Moreover, Kattou et al. (2013) identified a positive correlation between mathematical creativity and mathematical abilities, such as number sense, spatial ability, and inductive/deductive ability, and suggested conceptualising mathematical creativity as a subcomponent of mathematical ability.

As mathematical creativity can be enhanced by appropriate teaching methods (Hershkovitz et al., 2009), teachers and appropriate teaching environments are important in promoting mathematical creativity in school (Pitta-Pantazi et al., 2018). Teachers should first acknowledge the importance of creativity in enhancing mathematical understanding (Leikin, 2009a) and recognise how creativity connects to the mathematical curriculum (Boden, 2001); additionally, they may be able to promote mathematical creativity if they can identify creative behaviours and know which solution approaches can support these behaviours (Beghetto & Kaufman, 2009). Moreover, teachers should encourage students to think and inquire (Freiman, 2009), stimulate students’ interest and curiosity in creative investigation without constraining them with standard solutions (Mann, 2006), encourage them to seek various solutions (Achmetli et al., 2019; Presmeg, 2003), and allow them to express opinions freely and communicate with peers (Sriraman, 2009). Emotionally safe learning environments in which mistakes are not harshly criticised should be created in order to promote creativity (Sheffield, 2009). To reach these goals, mathematics teachers should adopt solution approaches different from those they were taught as students (Leikin & Elgrably, 2020). The promotion of creativity in school, teacher education, and professional development, is important (Klein & Leikin, 2020; Leikin & Elgrably, 2020). Teachers should themselves be able to investigate mathematics, be mathematically flexible, and be able to solve problems successfully, so that they can recognise their students’ creative solutions (Leikin et al., 2013). Departing from this state-of-the-art, in our study we investigate pre- and in-service teachers’ creative abilities to cope with mathematical modelling tasks, compared with the abilities of upper secondary school students who had experience in tackling mathematical modelling as a reference scheme.

### Mathematical creativity and mathematical modelling

The promotion of mathematical creativity is usually closely linked to problem-solving and problem-posing, both of which are often embedded in comprehensive theory-building processes (Assmus & Fritzlar, 2018). Generally, the development of creativity requires cognitively demanding mathematical tasks that entail high-level cognitive processes (Leikin & Elgrably, 2020). Mathematical modelling is characterised as being cognitively demanding; Niss and Højgaard (2011, 2019) emphasised cognitive abilities as being at the core of mathematical competencies. Moreover, the openness of modelling tasks makes modelling a creativity-directed activity since it requires and promotes mental flexibility and provides opportunities for the production of original ideas (Klein & Leikin, 2020; Wessels, 2014).

The mathematical modelling discourse is robustly intensifying worldwide, owing to its potentially significant contributions to citizenship education (Maaß et al., 2019). Mathematical modelling education promotes students’ abilities to solve real-world problems using modelling processes (Niss et al., 2007). Within the international discussion on teaching and learning of mathematical modelling competencies, this discourse has been shaped into four strands over the last two decades (for details, see Kaiser & Brand, 2015). Mathematical modelling competencies comprise both the ability and willingness to tackle real-world problems using mathematical methods, associated with affective issues, such as motivation and volition (Kaiser, 2017). Global modelling competencies are individuals’ abilities to perform and reflect on the entire modelling process successfully, and sub-competencies emphasise the individual phases of the modelling cycle, which varies with respect to the competencies necessary to complete the phases. Modelling cycles typically include the following sub-competencies linked to the phases of the modelling process (Kaiser, 2007; Maaß, 2006):

- Simplifying real-world problems and making adequate assumptions;
- Mathematising real-world problems;
- Tackling the mathematical model using adequate methods;
- Interpreting and validating the results, in the original real-world situation or even before in the real-world model.

Moreover, the modelling competencies construct involves general competencies such as the “competency to solve at least partly a real-world problem through mathematical description (that is, model) developed by oneself” (Kaiser, 2007, p. 111) and metacognitive competencies to make use of knowledge regarding modelling processes in general, to reflect on the modelling process and one’s own thinking (Stillman, 2011; Vorhölter, 2018). (For a more recent overview on the current discourse on modelling competencies see Cevikbas et al., 2021).

**Fig. 1:**
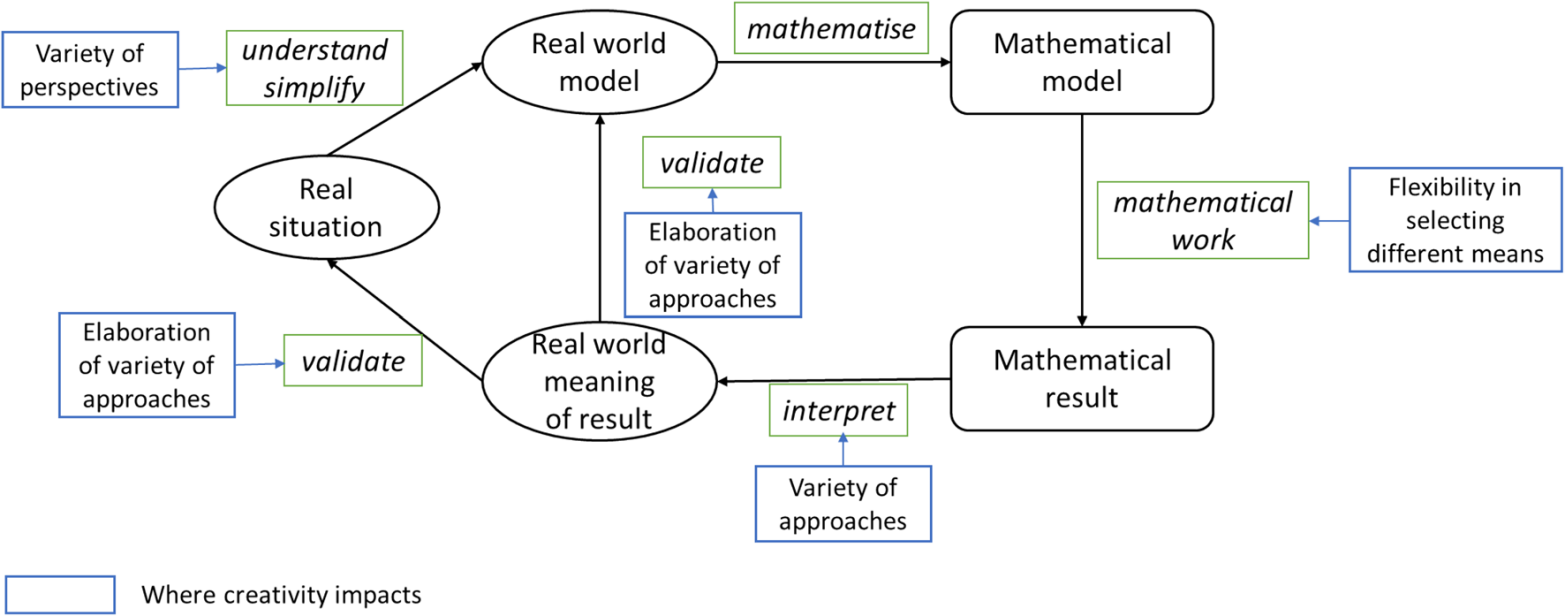
Modelling cycle enriched by aspects of creativity

Mathematical modelling requires creativity, which plays a key role in all phases of the modelling process driven by modelling competencies, and thus from the other side, the promotion of modelling competencies should also promote creativity. For example, mathematical modelling permits multiple solutions (Achmetli et al., 2019) and creative approaches, as usually no standard methods exist to solve underdetermined or unspecified, open real-world problems. The modelling process requires flexible and original ideas for its successful completion (Wessels, 2014). Specifically, creativity can provide a layered and unique understanding of real-world situations in analysing real-world problems. The flexible use of various mathematical means is necessary when developing mathematical solutions that reflect the value of varied mathematical content. More importantly, transverse ideas could be used to interpret and validate mathematical results, making sense of the results and linking them with new understandings of real-world situations based on the use of mathematical methods. As such, creativity should be integrated into the further development of the modelling competencies construct (Lu & Kaiser, 2021), with various creativity aspects embedded in the phases of the process, as represented by an enriched diagram (Fig. 1) originally provided by Kaiser and Stender (2013, p. 279).

### The evaluation of modelling competencies incorporating creativity

Modelling competencies and their development are highly related to the evaluation methods of the construct (Blum, 2015; Cevikbas et al., 2021; Niss & Blum, 2020). Evaluation of the abovementioned modelling competencies enriched by creativity should emphasise the components of creativity in the modelling process. Based on studies of general creativity (e.g., Torrance, 1966), researchers in mathematics education refined the components of mathematical creativity (e.g., Leikin, 2013; Silver, 1997). Consequently, the evaluation of three components—originality, fluency, and flexibility—has been broadly applied in studying mathematical creativity, particularly in the domains of problem-solving and problem-posing (Pitta-Pantazi et al., 2018).

Originality is usually considered the predominant characteristic of creativity (Pitta-Pantazi et al., 2018) and is included in numerous frameworks for the evaluation of creativity (Leikin, 2013; Reiter-Palmon et al., 2019). Moreover, it is suggested that originality determines creativity much more strongly than do the other two components of creativity (Levav-Waynberg & Leikin, 2012), and originality is described as a distinct, internal characteristic of creativity (Leikin, 2009b). Originality is usually defined by the newness and rareness of the corresponding responses, such as solutions to problems in problem-solving (Leikin, 2013) and the problems posed in problem-posing (Silver, 1997). Leikin (e.g., Leikin, 2009b, 2013) distinguishes relative originality from absolute originality: Relative originality is evaluated within a reference group, considering the reference norms, which relate strongly to the members’ previous experiences (Assmus & Fritzlar, 2018; Leikin, 2009b). This approach to evaluating originality has been widely applied in studying mathematical creativity (e.g., van Harpen & Siraman, 2013). Absolute originality is evaluated beyond the reference group and is usually studied at a global level through the inclusion of more reference groups, such as expert solvers in addition to young students (Leikin, 2013).

The evaluation of originality in mathematical modelling should include not only the originality of the mathematical means used, but also unique perspectives applied in interpreting real-world situations, which might generate multiple solutions to a problem (Lu & Kaiser, 2021). Expert solvers may employ mathematical means and heuristic strategies in modelling that go beyond school mathematics (Stender, 2017), but this does not mean that they can always show a higher level of originality in interpreting real-world situations than inexperienced solvers. It appears that evaluating originality in modelling is quite challenging, which cannot be justified simply by the educational trajectory or the level of education reached. Therefore, various reference groups should be included in the study of modelling creativity, considering different levels of education or different kinds of educational trajectories, which implies implications for the development of creativity in modelling. Based on this background, we included three groups in the study, as follows: upper secondary school students with experience in modelling, pre-service teachers with higher levels in mathematical learning, and in-service teachers with teaching experience.

Fluency and flexibility are also considered to be characteristics of creativity. Fluency evaluates the ability to generate as many ideas and approaches as possible to solve problems (Mann et al., 2017) and is usually scored based on the number of solutions offered (Leikin, 2013). Like originality, fluency is often supported by the idea that the higher the number of ideas proposed, the greater the possibility that an original idea will emerge (Pitta-Pantazi et al., 2018). Flexibility encourages different perspectives on and approaches to problems, to generate different ways of thinking (Mann et al., 2017; Pitta-Pantazi et al., 2018); consequently, it is usually evaluated based on the number of different solution types (Leikin, 2013). With a cognitive flexibility framework, Singer and Voica (2017) analysed the cognitive variety, cognitive novelty, and the capacity to make changes in cognitive framing. It appears that flexibility can be evaluated naturally based on the fluency and originality in the modelling, as each process includes several phases that may reveal a set of changes. Therefore, Lu and Kaiser (2021) suggested including only fluency as a creativity component in the evaluation of modelling competencies.

Usefulness is considered a unique component in the evaluation of creativity in modelling, considering the nature of modelling as applying mathematics in complex real-world problems (Lu & Kaiser, 2021). This approach differs from Sriraman’s (2009) conceptualisation of creativity in pure mathematics, whereby creative mathematical work may not always be applicable in other problems. Creativity in modelling should include usefulness, relevance, and adaptability; the reusability of modelling approaches in other real-world situations is thus included as an indicator of usefulness (Lu & Kaiser, 2021; Wessels, 2014).

As described above, we comprehensively discussed three creative components—originality, fluency, and usefulness—that can be included in the evaluation of creativity in modelling, based on the current creativity discourse in mathematics education and the understanding of the characteristics of modelling. These considerations contribute to the investigation of creativity perspectives in mathematical modelling in our study. Moreover, it is also necessary to examine the adequacy of modelling approaches, as described by the completeness and appropriateness of the processes. This analysis facilitates an independent reflection on participants’ modelling competencies and, based on the results, examines the relationship between modelling competencies and creative characteristics.

### Research context and questions

In this study we focus on the development of the construct of modelling competencies incorporating creativity, based on the study by Lu and Kaiser (2021), which was focusing on characteristics of upper secondary school students. Within their study, Lu and Kaiser (2021) developed a measurement instrument for students’ modelling competencies based on three modelling tasks, by focusing on the adequacy of the modelling processes manifested by the students, and the creative aspects of their modelling competencies in terms of usefulness, fluency and originality. For data evaluation, a coding manual was developed assessing these four criteria. It was found that although the students performed well on the easier task regarding adequacy, they did not perform well regarding creative aspects, especially originality; moreover, strong correlations were found among the four criteria, such as fluency and originality (Lu & Kaiser, 2021). In order to allow stronger insight into possible influential factors, this new study involved two more groups, namely pre- and in-service teachers, who had, in contrast to the school students, no experience in tackling modelling tasks, but were expected to have higher mathematical knowledge and richer experience in doing mathematics. Therefore, with this current study we aimed to explore whether the different experiences in mathematical modelling and mathematics would impact the participants’ manifestation of modelling competencies in terms of adequacy and the three creative aspects. Furthermore, we intended to find out, whether the correlations between the four criteria were consistent with the original findings involving only the student group. Specifically, the following two research questions were addressed:

1. Are there differences in the performance of the participants of three groups, with varying mathematical knowledge and modelling experience, on the three modelling tasks regarding the adequacy of the modelling approaches and the three aspects of creativity, namely usefulness, fluency, and originality?
2. Are the participants’ performances within the four criteria correlated to each other, especially concerning the three creative aspects, and if yes, how strongly?

## Methodology

### Participants and data collection

Data were collected from 50 pre-services teachers who were majoring in mathematics (Group 2) and 66 in-service mathematics teachers (Group 3) in addition to 71 upper secondary school students (Group 1). These participants comprised 100 females and 87 males (Table 1), with significant differences within the groups, which prohibited gender-related comparisons.

**Table 1:** Group distribution of the participants

	Group 1	Group 2	Group 3	Total
Female	14	38	48	100
Male	57	12	18	87
Total	71	50	66	187

The pre-service teachers were involved in the National Public-Funded Teacher Education Programme, which requires them to return to their own provinces to teach for several years. They were at the beginning of their third year of undergraduate studies, majoring in mathematics and applied mathematics. They had completed several mathematics courses (e.g., mathematical analysis and linear algebra) and general education courses (i.e., psychology and education) and had just commenced mathematical pedagogy courses. The 66 in-service teachers (for more information see Table 2), who were involved in a master’s programme in mathematics education, had been teaching for one year or more and were in the first year of their master’s studies. These pre- and in-service teachers had almost no previous experience in tackling modelling tasks. Differing from them, the school students, who aged 16–17 years, had experience of working on modelling tasks through attending modelling competitions. These 187 participants came from 21 of Mainland China’s 31 provinces.

**Table 2:** Distribution of teaching experience of the in-service teachers

	Primary school	Lower secondary school	Upper secondary school	Total
One to three teaching years	1	16	47	64
More than three teaching years	0	0	2	2
Total	1	16	49	66

The participants were asked to complete the three modelling tasks individually within one hour. They were encouraged to solve the tasks using different ways of solution. Their paperwork was collected as data for the study. The school students’ work on the three modelling tasks was collected in 2018 when they attended a summer school in modelling after having successfully attended a national modelling competition. Due to the COVID-19 pandemic, the in-service teachers completed the tasks during an online course in August 2020, and uploaded their paperwork within the allotted time. The pre-service teachers completed the tasks during an offline course on teaching and learning in mathematical modelling in early October, 2020. Since the pre- and in-service teachers had nearly no experience in modelling, they were given information on what mathematical modelling is, especially concerning the concept of the modelling cycle, which promotes modelling activities and plays an important role in the current curriculum.

### Measures

**Fig. 2:**
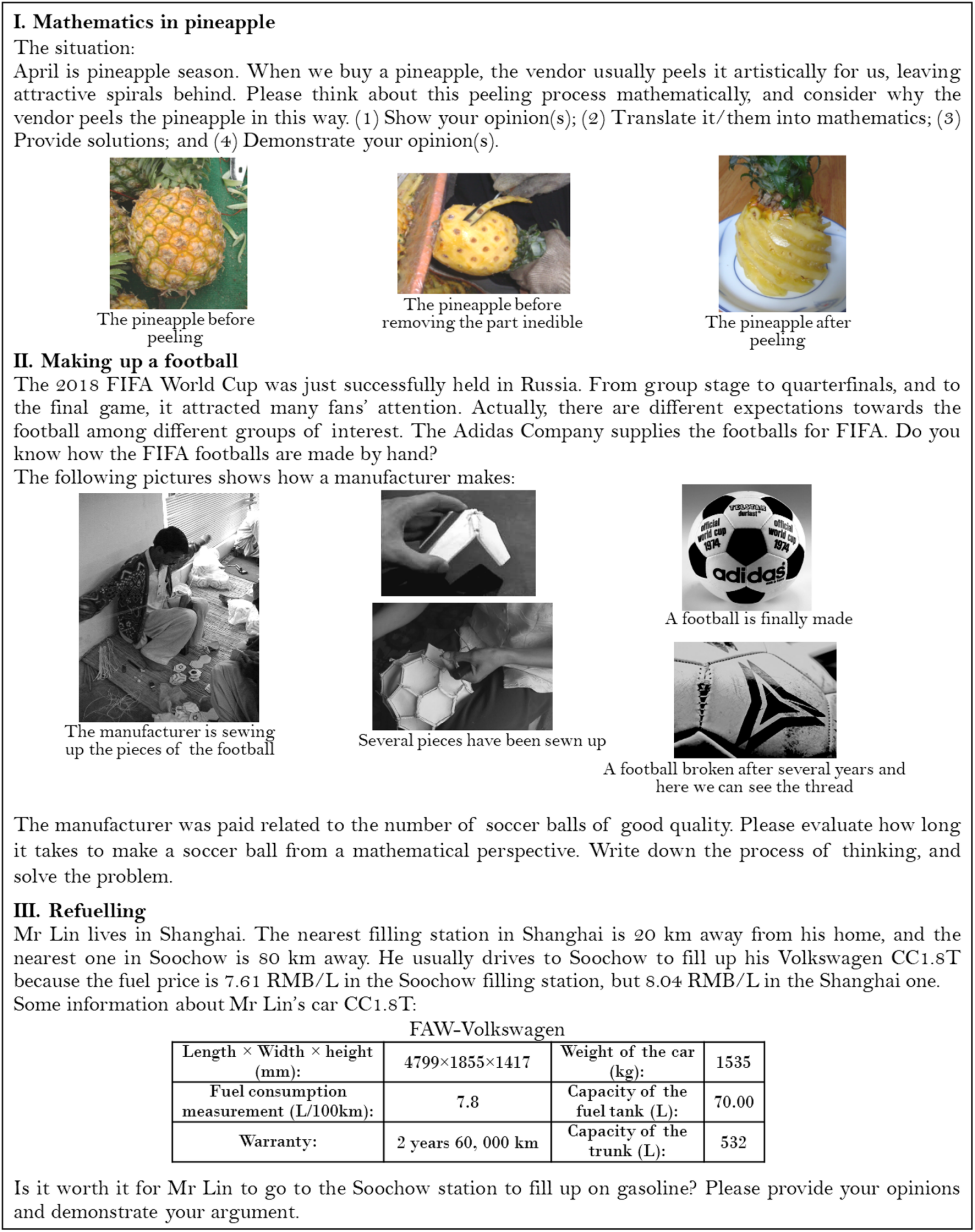
The three modelling tasks

In this study we followed Lu and Kaiser’s (2021) theoretical framework for measuring the participants’ modelling competencies through analysing their performance on the same three modelling tasks namely, *Peeling a pineapple*, *Making a World Cup Football*, and *Refuelling* (Fig. 2). Similar tasks had been used in earlier empirical studies on modelling competencies in Chinese and international contexts (e.g., Blum & Leiβ, 2005; Ludwig & Xu, 2009). These three tasks are varied with regard to their necessary or related mathematical knowledge, and the problem contexts. For example, *Refuelling* was considered to be a very familiar scenario for the participants, since similar tasks may have been tackled in school. These differences could have impacted the participants’ performance regarding their modelling competencies, especially creative aspects.

As mentioned above, for the study we firstly measured the participants’ modelling competencies in terms of the adequacy of the modelling approaches, considering whether the modelling approaches included completed modelling cycles that contain all steps of the modelling cycle (i.e., understanding and simplifying the tasks, building real-world and mathematical models, working within the mathematical model to obtain mathematical results, interpreting the results in the real-world context, and verifying whether the results resolve the real-world problem adequately). A three-level sub-category scheme for adequacy was used to grade the high–medium–low level of overall modelling competency, as follows:

- High—when the modelling approaches include relatively completed modelling cycles and are used to solve the task successfully;
- Medium—when the modelling approaches have the potential to be used to solve the tasks, but with uncompleted modelling cycles, (e.g., appropriate models have been built up but without mathematical results) or when the approaches include completed modelling cycles, but must be refined to solve the task successfully, for example by correcting mistakes in mathematical work on the model;
- Low—when the modelling approaches cannot be used to solve the task; for example, simply rephrasing the understanding of the task without a specific or acceptable model, or providing only mathematical results without a reproducible description of the process used.

Moreover, the three creative aspects—usefulness, fluency, and originality—were evaluated, using a three-level ordinal scale. Specifically, the evaluation addressed the following:

- Usefulness concerns the efficiency of a modelling approach in solving the task. A high level of usefulness is assigned to modelling approaches that are not only useful in solving the specific task but can also contribute to solving similar tasks; that is, the modelling approaches are shareable; a medium level is assigned to modelling approaches with the potential to solve the tasks but that cannot be reused; and a low level is assigned to modelling approaches that generate incorrect solutions.
- Fluency promotes the application of various solutions to the task. A high level of fluency is assigned to modelling approaches that include various models to solve tasks; a medium level indicates the modelling approaches include only one modelling cycle/model; and a low level indicates that no modelling cycle or an incomplete model is included in that approach.
- Originality concerns the modelling approach’s relative rarity, either in considering the parameters for building the models or the use of mathematical means to work within the models. A high level of originality indicates that the modelling approach includes important parameters and mathematical means that are used only by a small number of participants; a medium level indicates that the modelling approaches are used by a larger number of participants; and a low level shows that the approaches are used by most participants.

Details of the original coding can be found in the paper by Lu and Kaiser (2021); the adapted version of the coding manual referring to the adequacy of the modelling approaches and the subcategories of the creativity aspects is shown in the electronic supplementary material.

### Data analysis

We employed directed qualitative content analysis, which begins with existing research frameworks or findings––in our case, Lu and Kaiser’s (2021) coding manual––as the basis for the development of initial codes (Hsieh & Shannon, 2005). By means of this process, we first validated our understanding of the abovementioned adequacy of the modelling approach and the three aspects of creativity—usefulness, fluency, and originality. When coding with Lu and Kaiser’s (2021) coding scheme, which was developed based on the solutions to the three modelling tasks given by a group of upper secondary school students in China, some solutions could not be coded at first, and further analyses were needed before they could be categorised with the existing codes. For example, in the solutions to the football-making task, some participants used the interior angles of pentagons and hexagons to calculate the length of the common side, which could not be identified within the upper secondary school students’ work; these solutions were categorised as exhibiting high levels of usefulness and originality. Few changes were made to the coding manual, including the aspect of originality, because the rarity of the approaches did not change much in the pre-service and in-service teacher participants’ work on the three tasks, compared to that of the upper secondary school students.

Based on the coding, we analysed the participants’ performances in each task in terms of the approaches’ adequacy and creativity levels (including fluency, usefulness, and originality). A weighted kappa of $\ge 0.81$ shows a ‘very good’ inter-rater agreement on all the dimensions for each problem, according to Altman (1991, p. 404).

Next, Spearman correlation analysis was used to examine the relationship between participants’ performance on the four aspects (i.e., adequacy and the three creativity components), followed by partial correlation analysis to further identify the correlations between the three creativity aspects with a control of approach adequacy.

Third, a set of Friedman tests were used to compare participants’ performances across the three modelling tasks on each of the four aspects. When an overall significant difference was detected, Dunn-Bonferroni post hoc tests were used to further examine pairwise differences. Fourth, the between-group differences on each indicator were examined via a robust multivariate analysis of variance (MANOVA) using Munzel and Brunner’s (2000) method. Due to the sample’s uneven gender distribution, this study did not include any gender-related analyses. Finally, the partial credit model was used to generate the participants’ overall scores on the four performance indicators, and correlations among these scores were examined for the entire sample and each group.

## Results

We first present the results concerning the adequacy of the modelling approaches and the creativity aspects descriptively, addressing research question 1, before we describe the analyses of the correlations between the different dimensions, which addresses research question 2.

### Participants’ performance based on adequacy (research question 1)

Based on the whole group’s results (Table 3), it is noted that for Task 1, many of the participants performed at either a low or a high level of adequacy (49% or 46%, respectively). 50% of the group performed at a medium level and 34% performed at a high level on Task 2. Many participants built appropriate models but did not obtain mathematical or real-world answers. Of the participants, 94% performed at a high level of adequacy for Task 3. Based on the Friedman test, a significant difference was found in the participants’ modelling competencies across the three tasks, ${\chi }^{2}\left(2\right)=126.22, p<0.001$. Dunn-Bonferroni post hoc analysis revealed significant differences between Task 3 and Task 1 ($p<0.001$) as well as Task 2 ($p<0.001$) after Bonferroni adjustments. No difference was identified between Tasks 1 and 2 and the participants performed better on Task 3 than on Tasks 1 and 2.

**Table 3:** Percentages of participants at different adequacy levels across the three modelling tasks

Levels	Task 1	Task 2	Task 3
High	46%	34%	2%
Medium	5%	50%	4%
Low	49%	16%	94%

Using Munzel and Brunner’s (2000) method, the MANOVA on the ranked adequacy (i.e., participants’ performances, ranked among the three groups) revealed a significant difference across the three groups of participants, $F=5.54, p<0.001$ (Table 4). Further analysis revealed a significant difference between upper secondary school students (Group 1) and in-service teachers (Group 3), $F=10.70, p<0.001$, but no significant differences between upper secondary students (Group 1) and pre-service teachers (Group 2) and between pre-service (Group 2) and in-service teachers (Group 3). Table 4 indicates that for Group 1 the ranks were higher for Task 3 (0.53) than for Task 1 (0.41) and Task 2 (0.44), and for Group 3 the ranks were lower for Task 3 (0.47) than Task 1 (0.58) and Task 2 (0.53). It also shows for Tasks 1 and 2 that the ranks were higher for Group 3 than for Group 1, and for Task 3 that the ranks were higher for Group 1 than for Group 3.

**Table 4:** Typical ranks across groups on the three tasks regarding adequacy

	Task 1	Task 2	Task 3
Group 1	0.41	0.44	0.53
Group 2	0.46	0.49	0.51
Group 3	0.58	0.53	0.47

The descriptive results and the Friedman tests and Dunn-Bonferroni post hoc tests indicate that the participants performed better on Task 3 than Tasks 1 and 2 with respect to adequacy. Using the method of typical ranks (i.e., the average ranks across the combinations of groups) to describe the results, which indicate each group’s ranking, it appears that the upper secondary school students performed better on the easier task (Task 3), while the in-service teachers performed better on more difficult tasks (Tasks 1 and 2).

### The participants’ performance based on the creativity aspects (research question 1)

#### Usefulness

With respect to usefulness, 52% and 34% of the participants performed at a medium and low levels, respectively, on Task 1; 41% and 36% performed at high and low levels, respectively, on Task 2; and 59% and 39% performed at medium and high levels, respectively, on Task 3 (Table 5). A significant difference was noted between the tasks, ${\chi }^{2}\left(2\right)=50.52, p<0.001$. Further analysis revealed significant differences among all pairs of tasks: Tasks 1 and 2 ($p=0.006$), Tasks 1 and 3 ($p<0.001$), and Tasks 2 and 3 ($p=0.016$).

**Table 5:** Percentages of all participants at different usefulness levels across the three modelling tasks

Levels	Task 1 (%)	Task 2 (%)	Task 3 (%)
High	13	41	39
Medium	52	24	59
Low	34	36	2

Using Munzel and Brunner’s (2000) method for variance analysis, the MANOVA on the ranked usefulness reveals a significant difference across the three types of participants, $F=15.16, p<0.001$. Further analysis indicated significant differences between each pair of groups—Groups 1 and 3, $F=24.09, p<0.001$, Groups 1 and 2, $F=8.03, p<0.001$, and Groups 2 and 3, $F=5.30, p=0.001$. Table 6 indicates that the Group 1 ranks were lower for Task 2 (0.30) than for Task 1 (0.56) and Task 3 (0.54), the Group 2 ranks were higher for Task 3 (0.50) than Task 1 (0.44) and Task 2 (0.46), and the Group 3 ranks were higher for Task 2 (0.66) than Task 1 (0.49) and Task 3 (0.44). These results show that the ranks for Tasks 1 and 3 were higher for Group 1 than Group 3 and that the ranks for Task 2 were higher for Group 3 than Group 1.

**Table 6:** Typical ranks across groups on the three tasks regarding usefulness

	Task 1	Task 2	Task 3
Group 1	0.56	0.30	0.54
Group 2	0.44	0.46	0.50
Group 3	0.49	0.66	0.44

To summarise, the three groups of participants performed differently with respect to usefulness across the three tasks. Specifically, the upper secondary school students performed better on Tasks 1 and 3 than on Task 2, while the in-service teachers performed better on Task 2. These differences may be attributed to the in-service teachers’ more settled school mathematical knowledge—many figured out the number of edges and even the lengths of edges based on the mathematical relationships between pentagon and hexagon, which is school-level knowledge but requires strong transfer skills.

#### Fluency

Regarding fluency, most participants performed at a medium level (75%, 90%, and 96% for Tasks 1, 2, and 3, respectively), indicating that they presented one solution for the modelling tasks. On Task 1, 25% of the participants performed at a low level (Table 7). A significant difference was observed among the tasks ${\chi }^{2}\left(2\right)=55.48, p<0.001$, but in further analysis a significant difference was found only between Tasks 1 and 3 ($p=0.001$).

**Table 7:** Percentages of participants at different fluency levels across the three modelling tasks

Levels	Task 1 (%)	Task 2 (%)	Task 3 (%)
High	1	1	3
Medium	75	90	96
Low	25	9	2

The more detailed results concerning the variance, presented in Table 8, indicate no significant difference across the groups with respect to fluency ($F=0.68, p=0.60$), which is a quite unexpected result, as the experienced teachers could be expected to have an advantage.

**Table 8:** Typical ranks across groups on the three tasks regarding fluency

	Task 1	Task 2	Task 3
Group 1	0.48	0.48	0.51
Group 2	0.47	0.49	0.51
Group 3	0.50	0.51	0.48

#### Originality

Regarding originality, many participants performed at a low level—85%, 64%, and 72% on Tasks 1, 2, and 3, respectively. On Task 2, 19% of the participants performed at a high level, 11% performed at a high level on Task 1, and only 7% performed well on Task 3 (Table 9). A significant difference was observed across the tasks ${\chi }^{2}\left(2\right)=19.85, p<0.001$, and further analysis found a significant difference between Tasks 1 and 2 ($p=0.012$).

**Table 9:** Percentages of participants at different originality levels across the three modelling tasks

Levels	Task 1 (%)	Task 2 (%)	Task 3 (%)
High	11	19	7
Medium	4	17	21
Low	85	64	72

A significant difference was observed across the three groups with respect to originality, $F=12.62, p<0.001$. Further analysis indicated significant differences between Groups 1 and 3 ($F=20.46, p<0.001$) and between Groups 1 and 2 ($F=9.10, p<0.001$) and no significant difference between Groups 2 and 3. Table 10 indicates that the ranks of Group 1 were higher for Task 3 (0.66) than for Tasks 1 (0.54) and 2 (0.56), and for Groups 2 and 3 that ranks were lower for Task 3 than Tasks 1 and 2. The unexpected overall result is that for the three tasks, ranks were higher for Group 1 than for Groups 2 and 3.

**Table 10:** Typical ranks across groups on the three tasks regarding originality

	Task 1	Task 2	Task 3
Group 1	0.54	0.56	0.66
Group 2	0.50	0.50	0.47
Group 3	0.46	0.46	0.39

These results indicate that the upper secondary school students performed better with respect to originality than both pre- and in-service teachers and that they performed better on the easier than the difficult tasks in terms of originality; on the other hand, the in-service teachers in particular performed better on more difficult tasks (Tasks 1 and 2) than the easier task (Task 3) in terms of originality.

### Correlations between the dimensions (research question 2)

To address the second research question, we analysed the correlations among the adequacy of the modelling approaches and the three creativity aspects, and among the three creativity aspects themselves, using Spearman correlation analysis for each task (Table 11).

**Table 11:** Correlations among participants’ performance on different aspects

		Adequacy	Creativity-usefulness	Creativity-fluency	Creativity-originality
Task 1	Adequacy	1	0.719**	0.526**	− 0.105
	Creativity-usefulness		1	0.536**	0.036
	Creativity-fluency			1	0.257**
	Creativity-originality				1
Task 2	Adequacy	1	0.577**	0.335**	0.155
	Creativity-usefulness		1	0.340**	0.040
	Creativity-fluency			1	0.264**
	Creativity-originality				1
Task 3	Adequacy	1	0.098	0.264**	0.097
	Creativity-usefulness		1	0.060	0.393**
	Creativity-fluency			1	0.284**
	Creativity-originality				1

Note. **p < 0.01

Table 11 indicates significant correlations between adequacy and the creativity aspect of usefulness for Tasks 1 and 2, but much weaker correlations for Task 3. It also indicates significant correlations between adequacy and fluency on all the tasks, implying that the number of solutions is important for modelling adequacy. No significant correlations were identified between adequacy and originality.

Table 11 also indicates significant correlations between the creativity aspects, particularly between fluency and originality on all three tasks. Significant correlations were noted between usefulness and fluency on Tasks 1 and 2 and between usefulness and originality on Task 3 only. It appears that the significant correlation between usefulness and fluency is found on more difficult tasks, while the correlation between usefulness and originality is evident in easier tasks. Significant correlations remained when partial correlation analysis was conducted among the three aspects of creativity, controlling for adequacy, except for the correlation between usefulness and fluency on Task 2 (Table 12). This analysis identifies significant correlations between fluency and originality regardless of the type of task and the effect of adequacy, while the low correlations of the other two pairs of creativity aspects may be impacted by different types of tasks.

**Table 12:** Partial correlations among the creativity aspects

	Usefulness	Fluency	Originality
Task 1			
Usefulness	1	0.267**	0.161
Fluency		1	0.370**
Originality			1
Task 2			
Usefulness	1	0.190	− 0.062
Fluency		1	0.227**
Originality			1
Task 3			
Usefulness	1	0.036	0.387**
Fluency		1	0.270**
Originality			1

Note. **p < 0.01

Using the partial credit model, we generated the participants’ scores on all three tasks. Overall, significant correlations were found between adequacy and usefulness (${r}_{s}\left(187\right)=0.604, p<0.001$), adequacy and fluency (${r}_{s}\left(187\right)=0.466, p<0.001$), usefulness and fluency (${r}_{s}\left(187\right)=0.321, p<0.001$), and fluency and originality (${r}_{s}\left(187\right)=0.265, p<0.001$). Moreover, a significant correlation was also observed between usefulness and originality with ${r}_{s}\left(187\right)=-0.195, p=0.007$, which indicates that modelling approaches with higher levels of usefulness may imply lower levels of originality.

Concerning the overall scores, significant differences were noted based on $F$ tests in the three criteria (i.e., adequacy and the two creativity aspects of usefulness and originality) among all participants, except for fluency, consistent with the results reported in Sects. 4.1 and 4.2. The correlations between the four criteria differed among the different participant groups (Table 13). A significant strong correlation between adequacy and usefulness was noted in Group 1 (${r}_{s}\left(71\right),=0.697, p<0.001$), and a significant strong correlation between adequacy and fluency was found in Group 3 (${r}_{s}\left(66\right)=0.788, p<0.001$). In particular, the correlation between fluency and originality in Group 3 (${r}_{s}\left(66\right)=0.165, p=0.184$) was not significant as that in Group 1 (${r}_{s}\left(71\right),=0.370, p=0.002$) and Group 2 (${r}_{s}\left(50\right)=0.344, p=0.014$), and the correlation between usefulness and fluency in Group 1 (${r}_{s}\left(71\right)=0.278, p=0.019$) is not as significant as that in Group 2 (${r}_{s}\left(50\right)=0.402, p=0.004$) and 3 (${r}_{s}\left(66\right)=0.441, p<0.001$). These mixed results indicate that the correlations of these aspects may be influenced by the participants’ experiences in modelling and the richness of their mathematical knowledge.

**Table 13:** Correlations between the four criteria by the different groups of participants

	Group 1				Group 2				Group 3			
	Adequacy	Usefulness	Fluency	Originality	Adequacy	Usefulness	Fluency	Originality	Adequacy	Usefulness	Fluency	Originality
Adequacy	1	0.697**	0.348**	0.039	1	0.493**	0.282*	− 0.188	1	0.459**	0.788**	− 0.161
Usefulness		1	0.278*	0.081		1	0.402**	0.098		1	0.441**	− 0.163
Fluency			1	0.370**			1	0.344*			1	0.165
Originality				1				1				1

Note. *p < 0.05, **p < 0.01

## Summary, discussion and conclusion

### The participants’ performances on modelling and creativity

Many participants in the three groups—the upper secondary school students and pre-service and in-service teachers—provided adequate modelling approaches for the easiest modelling Task 3. For Task 1, the majority provided modelling approaches at either a low or a high level, indicating that the participants could present adequate modelling approaches when they had appropriate ideas. For Task 2, half of them provided medium-level modelling approaches in term of adequacy, meaning that the modelling approaches were appropriate to solve the modelling problem, but no complete solution was provided. These results are consistent with those from an international comparative study in China and Germany. Ludwig and Xu (2009) used a similar version of Task 1 to compare Chinese and German secondary students’ performances and found that the Chinese participants could produce adequate results for Task 1 if they could develop appropriate solution ideas. Furthermore, Chinese participants performed more poorly than their international counterparts on process-open tasks (Cai, 2000). These results highlight the strong emphasis on profound mathematical knowledge in the Chinese mathematical curriculum in recent decades.

The participants in this study did not perform well on the creativity aspects, particularly on originality and fluency. Most participants provided modelling approaches with low levels of originality on all three tasks. Only a few of the participants presented more than one approach for all tasks. This result is consistent with the related literature on mathematical creativity (e.g., van Harpen & Sriraman, 2013).

The participants performed differently across the three modelling tasks, and differences were observed among the participants within the three groups. Regarding the adequacy of the modelling approaches, the participants performed better on the easiest task (Task 3) than the other two. Further, the in-service teachers’ performances were significantly different from those of the upper secondary school students: the former performed better on difficult tasks than the latter, although the in-service teachers had no experience in modelling activities. This result was expected since the in-service teachers had richer mathematical knowledge and experience in applying mathematics. The result highlights the influence of mathematical knowledge in tackling complex modelling tasks.

However, this superiority in the adequacy of the modelling process was not mirrored in creativity. The upper secondary school students performed better than the pre- and in-service teacher groups on Tasks 1 and 3 in terms of usefulness, and they performed better than the other two groups on all three tasks in terms of originality, which is the predominant characteristic of creativity (Pitta-Pantazi et al., 2018). Although the development of new approaches to real-world problems requires sound mathematical knowledge (Niss & Blum, 2020), it appeared that the upper secondary school students had more original ideas than their adult counterparts, particularly when the mathematical knowledge needed to tackle the tasks did not go beyond school mathematics.

### The framework for evaluating creativity and modelling

Following the work of Lu and Kaiser (2021), this study demonstrated how modelling performance from the perspective of creativity can be evaluated, involving different participant groups with varying mathematical knowledge and teaching experience. With the participants’ varying characteristics in terms of mathematical knowledge, we examined the relationships between modelling and creativity and within the three creativity components—usefulness, fluency, and originality. We found significant correlations between the adequacy of modelling approaches and the three creativity components, particularly fluency and usefulness. The correlations between adequacy and originality were insignificant. Regarding the three creativity components, the correlations between usefulness and fluency appeared to be affected by adequacy—they decreased when controlling for adequacy—while the correlations between fluency and originality were consistently significant. These results are consistent with those in Lu and Kaiser’s (2021) study with upper secondary school students, and with other literature on the relationships between fluency and originality (e.g., Runco, 2010).

Considering the participants’ overall scores in the four criteria on the three modelling tasks, we found significant correlations between adequacy and the two creativity aspects of usefulness and fluency, which again confirms the significant correlations between modelling competencies and creativity. This result is in line with those of other studies, such as by Dan and Xie (2009), who also observed significant correlations between the modelling skills and creative thinking levels of university students. A significant negative correlation was found between usefulness and originality, which appears to be consistent with Sriraman’s (2009) view that original mathematical products may not always be applicable, and, that original approaches to real-world situations may not be largely generalisable. Further investigations are required to discuss in depth the relationships between usefulness and originality, referring to the specific nature and characteristics of mathematical modelling.

The results of the correlational analysis between the four criteria were not always consistent among the three participant groups. The correlation between usefulness and fluency was more significant in the groups of in- and pre-service teachers than among the upper secondary school students, which implies the effectiveness of the modelling approaches the pre- and in-service teachers could provide. However, on the other hand, the correlation between originality and fluency was insignificant in the group of in-service teachers, unlike that of the upper secondary school students. Moreover, the correlation between usefulness and originality was negative in the group of in-service teachers. These results indicate that the developed framework on mathematical modelling enriched by creativity should take into account the variety of the characteristics of the groups.

Overall, further research is necessary to explore the relationships among the creativity components, and the factors that are influential in the relationships. Moreover, considering the significant differences in the participants’ performances across the three tasks, the development of the framework should include the investigation of a greater variety of modelling tasks.

### Mathematical modelling as a creativity-demanding activity

Due to its openness and process-oriented characteristics, mathematical modelling has the potential to be a creativity-demanding activity. The present study demonstrated how such an activity could be evaluated by examining creative aspects in the performance of modelling processes, during which both global modelling competencies and sub-competencies of mathematical modelling are reflected (Lu & Kaiser, 2021). The study’s results indicate a significant correlation between modelling competencies and creativity aspects, suggesting that modelling may be conceptualised as a creativity-demanding activity. In particular, the results of the significant correlations provide insights for the promotion of both creativity and modelling. Furthermore, the results, which considered the differences among the three groups of participants’ performances on the modelling tasks with different difficulty levels, have implications for the promotion of creativity for various groups and demonstrate the dependency of the modelling activities on the mathematical knowledge of the participants and the mathematical topic with which they dealt. That means, a modelling task as a creativity-demanding activity should be situated in the proximal range of the person’s expertise, but going beyond this level of expertise at a certain point, as suggested by Singer and Voica (2017) to promote the relevant level of expertise. Furthermore, due to the self-differentiating potential of mathematical modelling problems (Borromeo Ferri, Kaiser, & Paquet, under review), and their openness and potential for mathematical insight, each modelling problem can serve to a certain extent as a creativity-directed activity (Leikin, 2018). However, empirical studies will be needed in order to examine this claim. Overall, the study points out that students and teachers, both pre- and in-service teachers, need explicit opportunities to develop their own creativity skills through tackling creativity-demanding modelling tasks on their own.

This study has some limitations. The first is the small number of modelling tasks, which may influence the results considerably. As became evident, the results concerning the adequacy of the modelling approach and the creativity dimensions were strongly influenced by the tasks used. Therefore, a study using a higher variety of modelling tasks may potentially broaden and substantiate the results achieved. Furthermore, the fact that the samples were convenience samples constitutes a considerable limitation that should be resolved in future studies.

Overall, a stronger variability in the modelling tasks and a stronger representativeness of the samples used is highly desirable, and would allow the overcoming of certain weaknesses of the study. However, this study is among the first to integrate creativity into modelling processes, offering insight into connections between different fields of mathematics education and in particular into different aspects of mathematical thinking, a highly important field of research within mathematics education, independent of mathematical modelling.
